# Supplementary material for: The intracellular bacterium Orientia tsutsugamushi uses the autotransporter ScaC to activate BICD adaptors for dynein-based motility
Source: Nat Commun. 2025 Jul 3;16:6122. doi: 10.1038/s41467-025-61105-5 (PMC12229497; doi:10.1038/s41467-025-61105-5)
Supplement: Supplementary file 1 — Supplementary Information [file 41467_2025_61105_MOESM1_ESM.pdf]

SUPPLEMENTARY FIGURES

The intracellular bacterium Orientia tsutsugamushi uses the autotransporter ScaC to activate BICD adaptors for dynein-based motility.

Manigrasso *et al*.

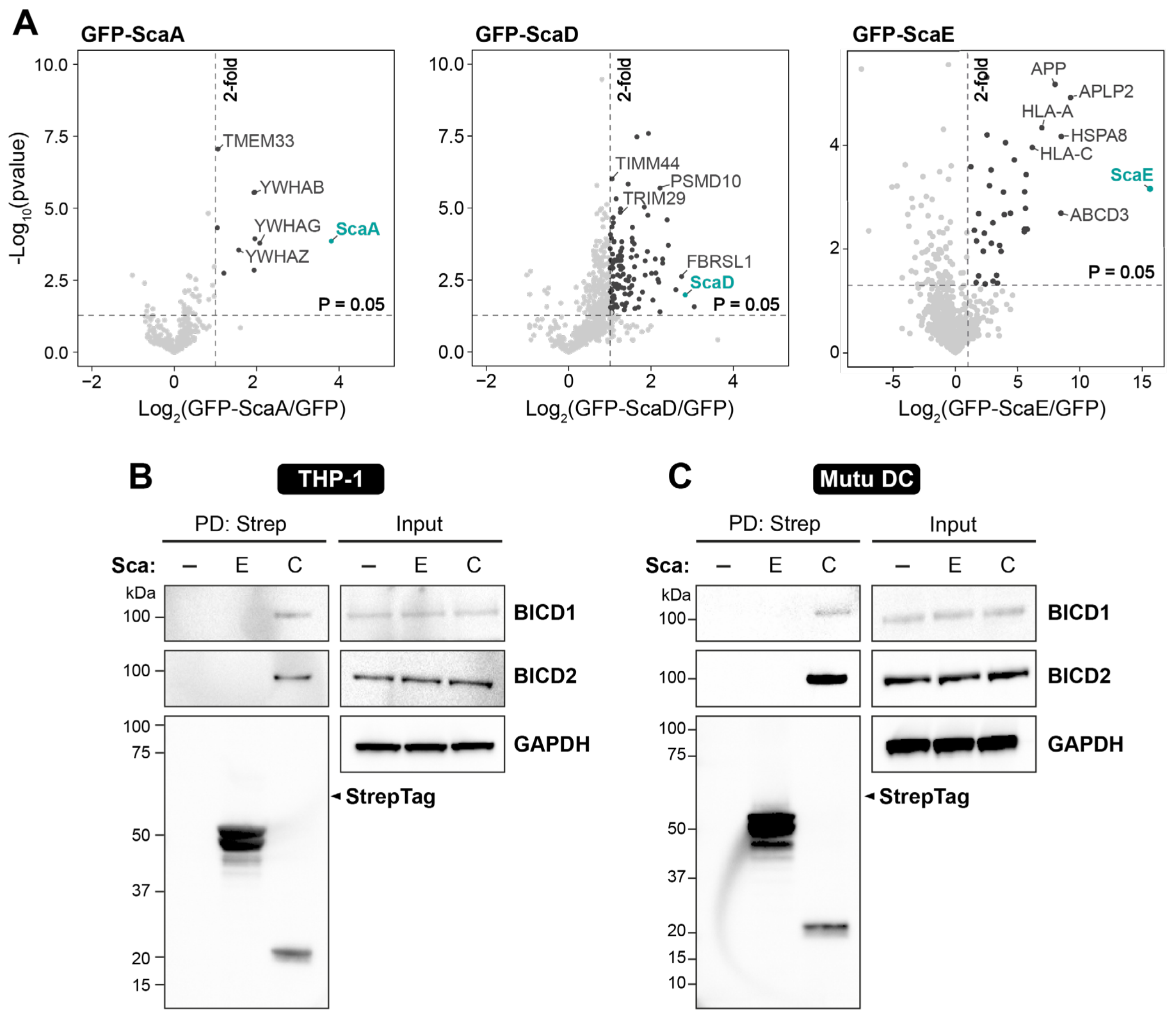

Supplementary Figure 1. ScaC binds the activating adaptors BICD1 and BICD2 in different cell types. (A) Volcano plots summarising the results of GFP-ScaA, GFP-ScaD and GFP-ScaE co-IP/MS in HeLa Flp-In cells. The significant enrichment levels were calculated from n=3 independent replicates using a two-sided t-test. Selected hits are indicated. **(B)** and **(C)** show the results of Strep pull-down experiments with monocyte (THP-1) and dendrite (MutuDC) cell line lysates (respectively) using purified Strep-tagged ScaE (E) or ScaC (C) protein as baits. Pull-down experiments were repeated twice. Source data are provided as a Source Data file.

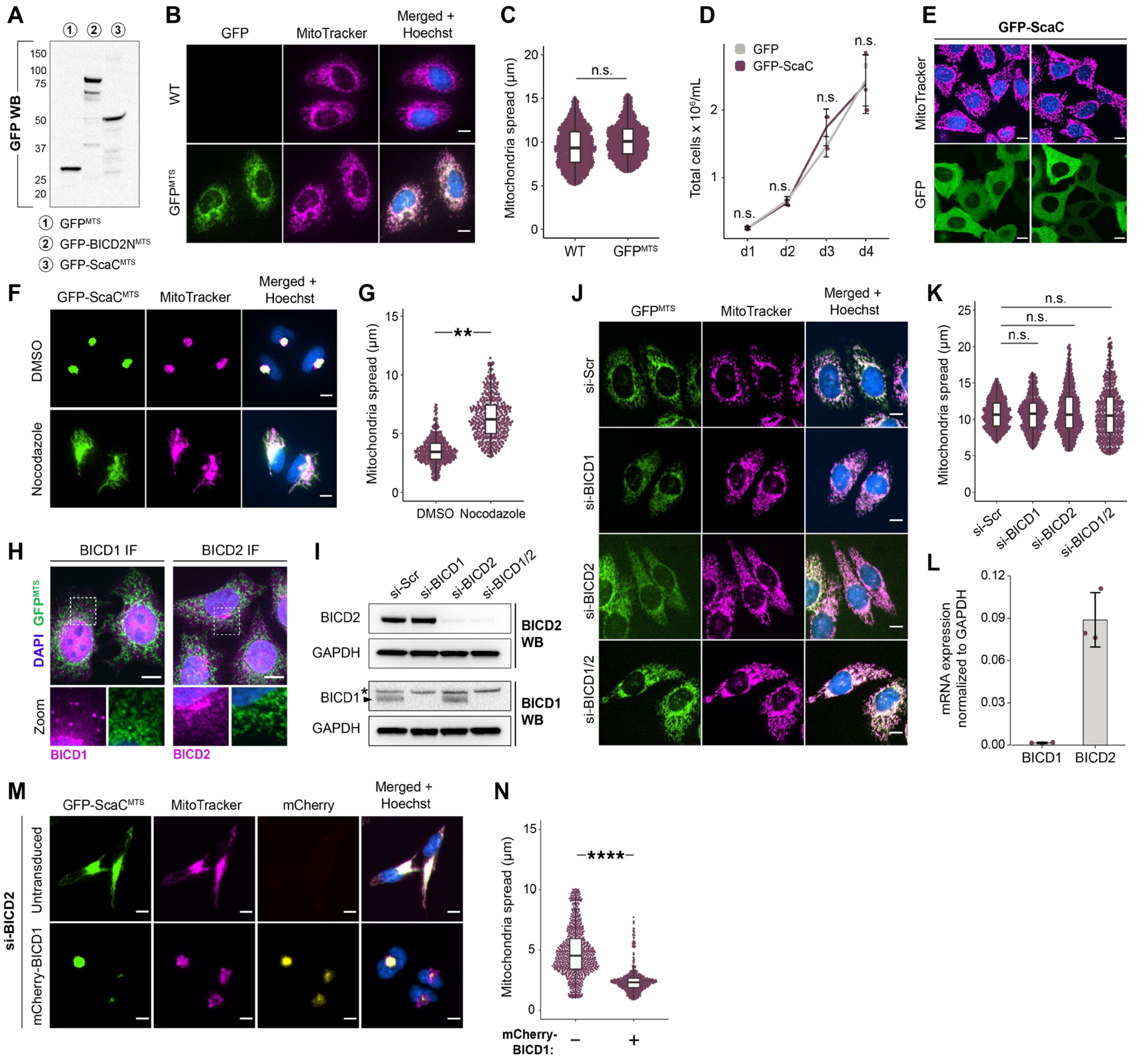

Supplementary Figure 2. ScaC is sufficient to elicit dynein-driven movement in cells. (A) Western blot showing expression of GFP^{MTS}, GFP-BICD2N^{MTS} and GFP-ScaC^{MTS} in Hela Flp-In cells. **(B)** Representative images of mitochondrial relocation assay with wild-type and GFP^{MTS} cell lines. Scale bars indicate 10 µm. The corresponding quantification of mitochondrial spread is shown in (**C**). P-values were calculated from n=3 independent replicates using two-tailed ANOVA and a Tukey HSD post-hoc test. **(D)** Lineplot showing the growth of GFP and GFP-ScaC HeLa Flp-In cells in the presence of tetracycline over a periof of four days. Error bars indicate mean ± standard deviation. P-values were calculated from n=3 independent replicates using a two-tailed T-test. **(E)** Representative images showing the distribution of mitochondria (magenta) in GFP-ScaC cells. Scale bars represent 10 µm. **(F)** Representative images of GFP-ScaC^{MTS} cells treated with either DMSO or 10 µm nocodazole for 3 hours. The corresponding quantification of mitochondrial spread is shown in **(G).** P-values were calculated from n=3 independent replicates using two-tailed ANOVA and a Tukey HSD post-hoc test. ***=p<0.001. **(H)** Representative IF images showing a lack of clear colocalization of GFP^{MTS} with BICD1 and BICD2 respectively. Scale bars indicate 5 µm. **(I)** Western blots showing knockdown of BICD1 and BICD2 upon siRNA treatment. GAPDH was used as a loading control. The asterisk indicates a non-specific GAPDH band. **(J)** Representative images of GFP^{MTS} cells treated with siRNA against BICD1 and/or BICD2. A scrambled siRNA (si-Scr) was used as a negative control. Scale bars indicate 10 µm. **(K)** Quantification of mitochondrial spread in siRNA-treated GFP^{MTS} cells. P-values were calculated from n=3 independent replicates using two-tailed ANOVA and a Tukey HSD post-hoc test. **(L)** Barplot showing mRNA expression of *BICD1* and *BICD2* relative to *GAPDH* in HeLa Flp-In GFP-ScaC^{MTS} cells. Error bars indicate mean ± standard deviation. **(M)** Representative images of mitochondrial relocation assay with BICD2-depleted GFP-ScaC^{MTS} cells in the presence of absence of a mCherry-BICD1 overexpression cassette. Scale bars indicate 10 µm. The corresponding quantification of mitochondrial spread is shown in (**N).** P-values were calculated from n=3 independent replicates using two-tailed T-test. ****=p<0.0001. In (C), (G), (E) and (N) the box plot centres indicate the median, the boxes show the 25th/75th percentile, and whiskers extend to the largest or smallest values within 1.5×IQR from the hinges. Source data are provided as a Source Data file.

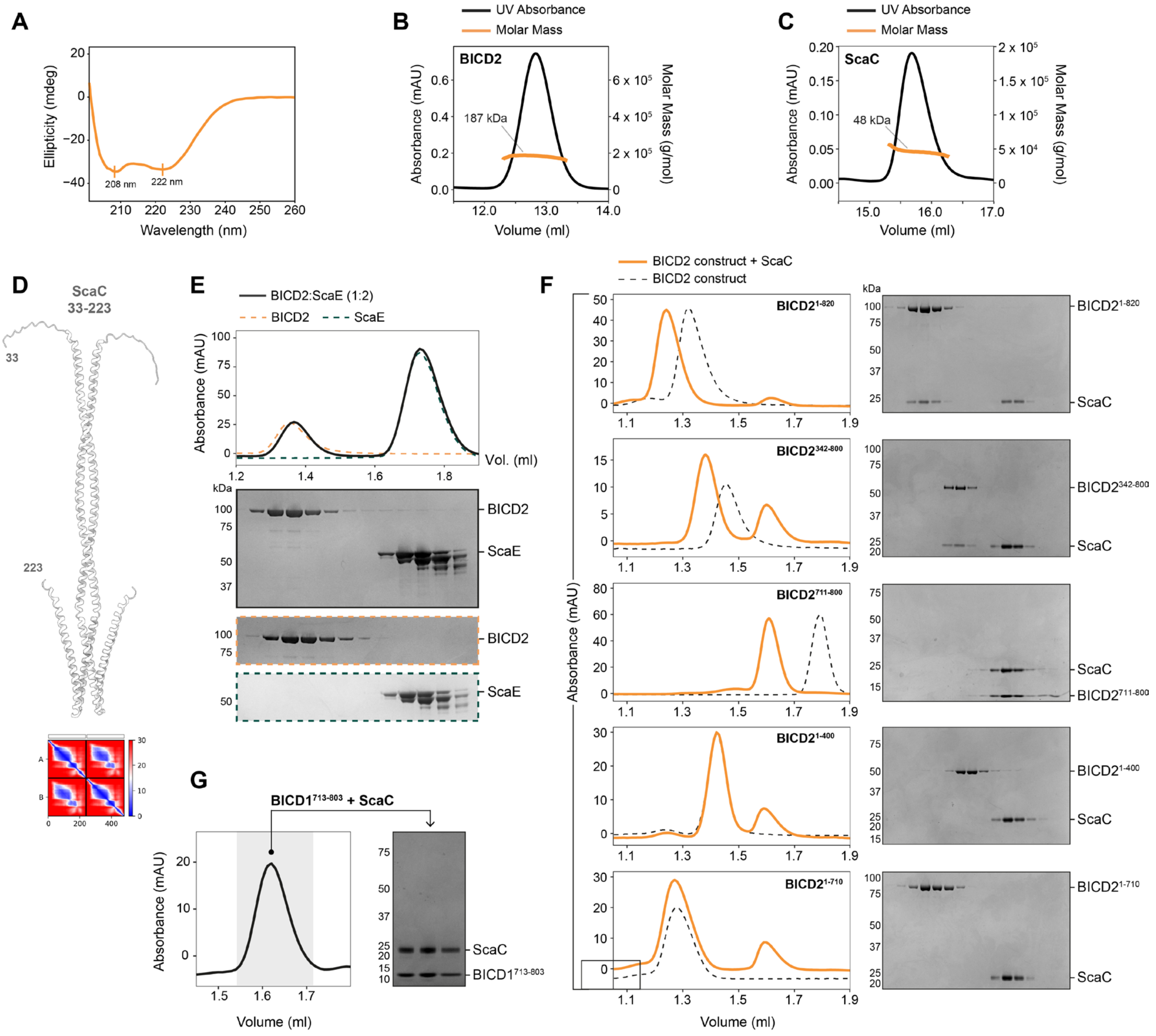

Supplementary Figure 3. ScaC binds the C-terminal CC3 domain of BICD2. (A) Circular dichroism analysis of purified ScaC. The plot shows ellipticity (mdeg) as a function of wavelength (nm), and the secondary structure of the protein is inferred from the shape and magnitude of the ellipticity curve. The annotated dips at 208 nm and 222 nm suggest that the passenger domain of ScaC is predominantly alpha helical. **(B-C)** SEC-MALS of purified BICD2 and ScaC respectively. Molecular masses are indicated for both proteins. **(D)** Alphafold prediction of the passenger domain of ScaC (residues 33-223). **(E)** Chromatograms showing BICD2 in the presence (black) or absence (orange) of ScaE. The SDS-PAGE gels show the fractions underlying the relevant protein peaks. **(F)** Chromatograms showing ScaC in complex with different BICD2 truncations. Dotted lines represent chromatographs of each BICD2 construct in the absence of ScaC. SDS-PAGE gels show fractions underlying the relevant protein peaks. **(G)** Chromatogram showing BICD1$^{713\text{-}803}$ in complex with ScaC. The SDS-PAGE gel shows the fractions underlying the complex peak. All SEC experiments were performed three times. Source data are provided as a Source Data file.

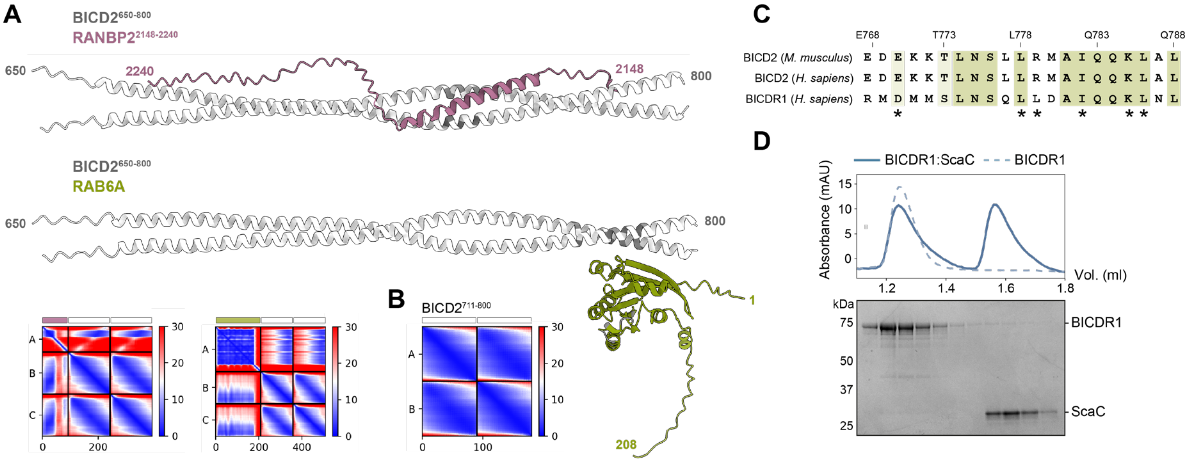

Supplementary Figure 4. ScaC binds BICD2 through a novel interface . (A) AlphaFold predictions of BICD2$^{650\text{-}800}$ in complex with either RANBP2$^{2148\text{-}2240}$ (top) or RAB6A (bottom). Biochemically validated interface residues are highlighed in grey. PAE plots are shown. **(B)** PAE blot for the AlphaFold prediction of BICD2$^{711\text{-}800}$ shown in Figure 3F. **(C)** Multiple sequence alignment of the RAB6-binding site across mouse BICD2, human BICD2 and human BICDR1. Residue numbers refer to murine BICD2. Asterisks indicate residues required for the interaction with RAB6. **(D)** Chromatogram showing BICDR1 in the presence (solid line) or absence (dotted line) of ScaC. SDS-PAGE gels show fractions underlying the relevant protein peaks. The SEC experiment was performed twice. Source data are provided as a Source Data file.

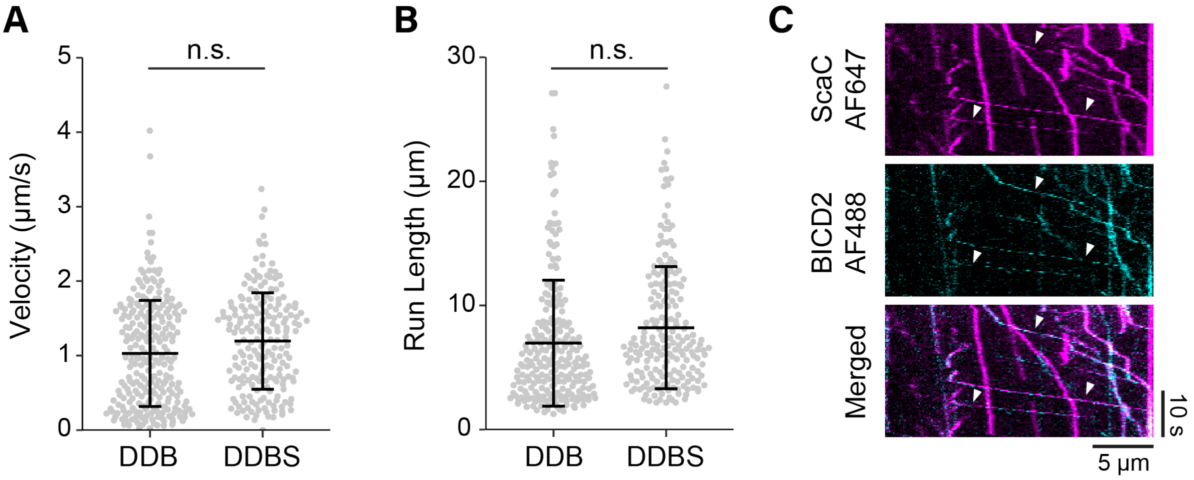

Supplementary Figure 5. ScaC activates BICD2 for dynein motility. (A) Quasi-random scatter plot showing velocity of DDB and DDBS complexes. The centre line shows the mean and the whiskers 1 standard deviation. P-values were calculated from n=3 independent replicates using a two-tailed T- test. **(B)** Quasi-random scatter plot showing run length of DDB and DDBS complexes. The centre line shows the mean and the whiskers 1 standard deviation. P-values were calculated from n=3 independent replicates using a two-tailed T- test. **(C)** Representative kymographs showing colocalization of Scac-AF674 (magenta) and BICD2-AF488 (cyan). Arrows indicate colocalization events. Scale bars are shown. Source data are provided as a Source Data file.

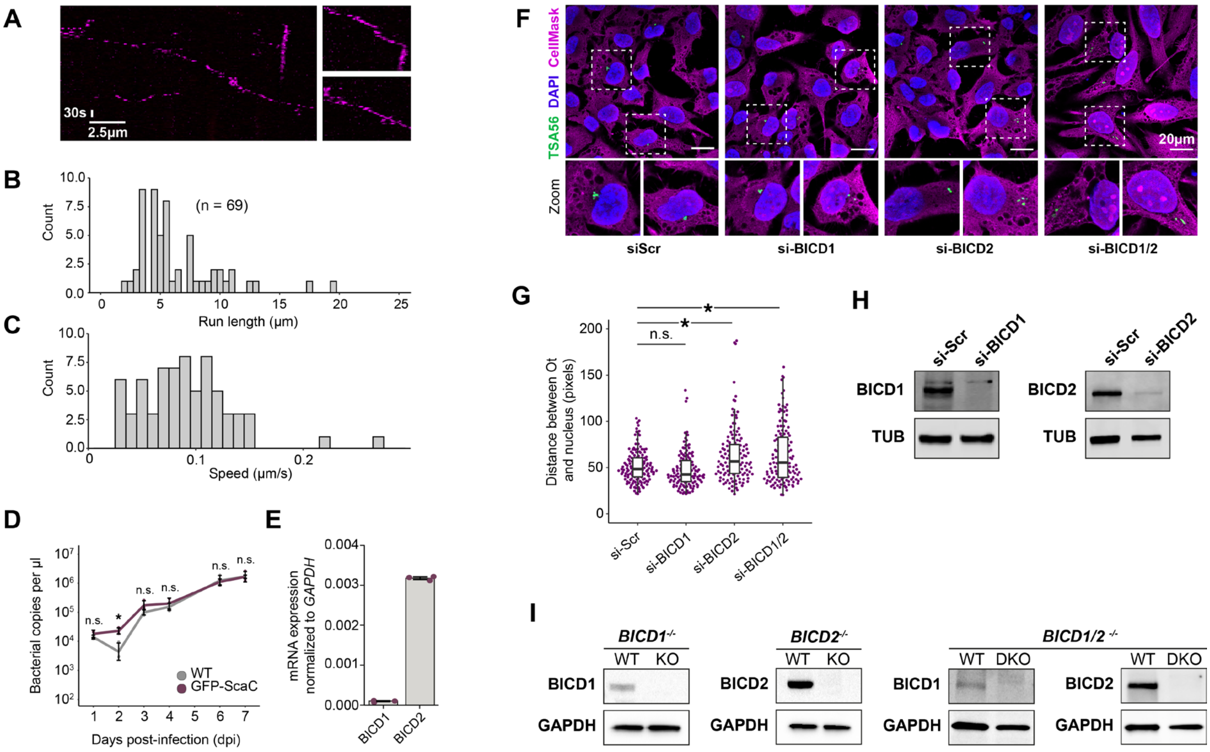

Supplementary Figure 6. BICD1 and BICD2 are required for perinuclear transport of *O. tsutsugamushi*. (A) Representative kymographs showing the movement of *O. tsutsugamushi* in L929 cells at 4-5 hpi. **(B)** Histogram of the run lengths of *O. tsutsugamushi* transport at 4-5 hpi. **(C)** Histogram of the speeds of O.tsutsuamushi transport at 4-5 hpi **(D)** Lineplot showing the bacterial copy number per ul of sample in wild-type (WT) vs. GFP-ScaC expressing HeLa ATCC CCL-2 cells at different times post-infection. Error bars indicate mean ± standard deviation. P-values were calculated from n=3 independent replicates using a two-tailed T-test. *=p<0.05. **(E)** Barplot showing mRNA expression of *BICD1* and *BICD2* relative to *GAPDH* in HeLa ATCC CCL-2 cells. Error bars indicate mean ± standard deviation. **(F)** Representative IF images showing localization of *O. tustsugamushi* (green) at 24 hpi in HeLa ATCC CCL-2 cells treated with siRNAs against BICD1 and/or BICD2. A scrambled siRNA (si-Scr) was used as a negative control. **(G)** Quantification of the distance between bacteria and the nucleus in siRNA-treated HeLa ATCC CCL-2 cells. The beeswarm plot includes all values between 5th and 95th percentiles. The box plot centre indicates the median, the boxes show the 25th/75th percentile, and whiskers extend to the largest or smallest values within 1.5×IQR from the hinges. P-values were calculated from n=3 independent replicates using two-tailed ANOVA (F value = 10.948) with a Tukey HSD post-hoc test. *=p<0.05. **(H)** Western blots showing knockdown of BICD1 and BICD2 upon siRNA treatment. B-tubulin was used as a loading control. **(I)** Western blot validation of BICD1, BICD2 knockouts (KO) and a BICD1/2 double knockout (DKO) in HeLa ATCC cells. Source data are provided as a Source Data file.
