# Supplementary material for: The intracellular bacterium Orientia tsutsugamushi uses the autotransporter ScaC to activate BICD adaptors for dynein-based motility
Source: Nat Commun. 2025 Jul 3;16:6122. doi: 10.1038/s41467-025-61105-5 (PMC12229497; doi:10.1038/s41467-025-61105-5)
Supplement: Supplementary file 2 — Description of Additional Supplementary Files [file 41467_2025_61105_MOESM2_ESM.pdf]

Description of Additional Supplementary Files:

Supplementary Data 1: Mass spectrometry hits for GFP-ScaA co-IP. List of proteins specifically coprecipitating with GFP-ScaA. MW = molecular weight, FC = log2(Fold Change), P-Value = -log10(p-value). Pvalues were calculated from triplicate runs using a two-sided t-test. Peptide identifications were filtered for high confidence using the Percolator node (FDR < 1%, rank 1 peptides).

Supplementary Data 2: Mass spectrometry hits for GFP-ScaD co-IP. List of proteins specifically coprecipitating with GFP-ScaD. MW = molecular weight, FC = log2(Fold Change), P-Value = -log10(p-value). Pvalues were calculated from triplicate runs using a two-sided t-test. Peptide identifications were filtered for high confidence using the Percolator node (FDR < 1%, rank 1 peptides).

Supplementary Data 3: Mass spectrometry hits for GFP-ScaE co-IP. List of proteins specifically coprecipitating with GFP-ScaE. MW = molecular weight, FC = log2(Fold Change), P-Value = -log10(p-value). Pvalues were calculated from triplicate runs using a two-sided t-test. Peptide identifications were filtered for high confidence using the Percolator node (FDR < 1%, rank 1 peptides).

Supplementary Data 4: Mass spectrometry hits for GFP-ScaC co-IP. List of proteins specifically coprecipitating with GFP-ScaC. BICD1 and BICD2 are highlighted. MW = molecular weight, FC = log2(Fold Change), P-Value = -log10(p-value). P-values were calculated from triplicate runs using a two-sided t-test. Peptide identifications were filtered for high confidence using the Percolator node (FDR < 1%, rank 1 peptides).

Supplementary Data 5: Crosslnking mass spectrometry results for the BICD2-ScaC complex. Crosslinked residues for BICD2 and ScaC and their respective match scores are indicated. Crosslinks within the CC3 domain of BICD2 (residues 711-800) are highlighted.

Supplementary Movie 1: Representative movie showing live movement of *O. tsutsugamushi*, labelled with CellTrace™ CFSE, in L929 cells at 4 hpi. Scale bar is 5 μm. Frames were collected every 5.8s.
