# Supplementary material for: The intracellular bacterium Orientia tsutsugamushi uses the autotransporter ScaC to activate BICD adaptors for dynein-based motility
Source: Nat Commun. 2025 Jul 3;16:6122. doi: 10.1038/s41467-025-61105-5 (PMC12229497; doi:10.1038/s41467-025-61105-5)
Supplement: Supplementary file 9 — Reporting Summary [file 41467_2025_61105_MOESM9_ESM.pdf]

Corresponding author(s): Andrew P Carter, Jeanne Salje

Last updated by author(s): 2025/02/18

Reporting Summary

Nature Portfolio wishes to improve the reproducibility of the work that we publish. This form provides structure for consistency and transparency in reporting. For further information on Nature Portfolio policies, see our Editorial Policies and the Editorial Policy Checklist.

Statistics

For all statistical analyses, confirm that the following items are present in the figure legend, table legend, main text, or Methods section.

n/a	Confirmed	
☐	☒	The exact sample size (*n*) for each experimental group/condition, given as a discrete number and unit of measurement
☐	☒	A statement on whether measurements were taken from distinct samples or whether the same sample was measured repeatedly
☐	☒	The statistical test(s) used AND whether they are one- or two-sided<br>*Only common tests should be described solely by name; describe more complex techniques in the Methods section.*
☐	☒	A description of all covariates tested
☒	☐	A description of any assumptions or corrections, such as tests of normality and adjustment for multiple comparisons
☐	☒	A full description of the statistical parameters including central tendency (e.g. means) or other basic estimates (e.g. regression coefficient) AND variation (e.g. standard deviation) or associated estimates of uncertainty (e.g. confidence intervals)
☐	☒	For null hypothesis testing, the test statistic (e.g. *F*, *t*, *r*) with confidence intervals, effect sizes, degrees of freedom and *P* value noted<br>*Give P values as exact values whenever suitable.*
☒	☐	For Bayesian analysis, information on the choice of priors and Markov chain Monte Carlo settings
☒	☐	For hierarchical and complex designs, identification of the appropriate level for tests and full reporting of outcomes
☒	☐	Estimates of effect sizes (e.g. Cohen's *d*, Pearson's *r*), indicating how they were calculated

Our web collection on statistics for biologists contains articles on many of the points above.

Software and code

Policy information about availability of computer code

Data collection	NIS Elements (version 5.1)<br>NIS Elements (version 5.4)<br>µManager (version 1.4.22)<br>Leica LAS X software (version 4)<br>ZEN 2011 SP7 FP3 (version 14.0.0.0)
Data analysis	Fiji ImageJ Software version 2.8.0<br>RStudio version 4.3.3<br>Cell organization Macro: https://github.com/jboulanger/imagej-macro/tree/main/Cell_Organization<br>Cellprofiler version 4.2.6

For manuscripts utilizing custom algorithms or software that are central to the research but not yet described in published literature, software must be made available to editors and reviewers. We strongly encourage code deposition in a community repository (e.g. GitHub). See the Nature Portfolio guidelines for submitting code & software for further information.

Data

Policy information about availability of data

All manuscripts must include a data availability statement. This statement should provide the following information, where applicable:

- Accession codes, unique identifiers, or web links for publicly available datasets
- A description of any restrictions on data availability
- For clinical datasets or third party data, please ensure that the statement adheres to our policy

Source data (except microscopy images) are provided in 2 uploaded .xlsx files: Source_data_main_figures.zip and Source_data_supplemental.zip
All raw data including microscopy images are uploaded at: 10.5281/zenodo.14888115

Research involving human participants, their data, or biological material

Policy information about studies with human participants or human data. See also policy information about sex, gender (identity/presentation), and sexual orientation and race, ethnicity and racism.

Reporting on sex and gender	*Use the terms sex (biological attribute) and gender (shaped by social and cultural circumstances) carefully in order to avoid confusing both terms. Indicate if findings apply to only one sex or gender; describe whether sex and gender were considered in study design; whether sex and/or gender was determined based on self-reporting or assigned and methods used. Provide in the source data disaggregated sex and gender data, where this information has been collected, and if consent has been obtained for sharing of individual-level data; provide overall numbers in this Reporting Summary. Please state if this information has not been collected. Report sex- and gender-based analyses where performed, justify reasons for lack of sex- and gender-based analysis.*
Reporting on race, ethnicity, or other socially relevant groupings	*Please specify the socially constructed or socially relevant categorization variable(s) used in your manuscript and explain why they were used. Please note that such variables should not be used as proxies for other socially constructed/relevant variables (for example, race or ethnicity should not be used as a proxy for socioeconomic status). Provide clear definitions of the relevant terms used, how they were provided (by the participants/respondents, the researchers, or third parties), and the method(s) used to classify people into the different categories (e.g. self-report, census or administrative data, social media data, etc.) Please provide details about how you controlled for confounding variables in your analyses.*
Population characteristics	*Describe the covariate-relevant population characteristics of the human research participants (e.g. age, genotypic information, past and current diagnosis and treatment categories). If you filled out the behavioural & social sciences study design questions and have nothing to add here, write "See above."*
Recruitment	*Describe how participants were recruited. Outline any potential self-selection bias or other biases that may be present and how these are likely to impact results.*
Ethics oversight	*Identify the organization(s) that approved the study protocol.*

Note that full information on the approval of the study protocol must also be provided in the manuscript.

Field-specific reporting

Please select the one below that is the best fit for your research. If you are not sure, read the appropriate sections before making your selection.

☒ Life sciences ☐ Behavioural & social sciences ☐ Ecological, evolutionary & environmental sciences

For a reference copy of the document with all sections, see nature.com/documents/nr-reporting-summary-flat.pdf

Life sciences study design

All studies must disclose on these points even when the disclosure is negative.

Sample size	The microscopy sample size is 25-30 images/condition/replicate and we have triplicate datasets.
Data exclusions	No data was excluded from the analysis
Replication	The number of independent replicates was always >3, as specified in the figure legends
Randomization	For microscopy experiments, the imaging fields were randomly chosen for all conditions. To ensure the presence of cells in our images, the fields of view were selected using CellMask or Hoechst staining, which were largely unaffected by the conditions tested in our experiments. These fields were then images with the fluorescence channels of interest.
Blinding	Blinding was not carried out in this study.

Reporting for specific materials, systems and methods

We require information from authors about some types of materials, experimental systems and methods used in many studies. Here, indicate whether each material, system or method listed is relevant to your study. If you are not sure if a list item applies to your research, read the appropriate section before selecting a response.

Materials & experimental systems

n/a	Involved in the study	
☐	☒	Antibodies
☐	☒	Eukaryotic cell lines
☒	☐	Palaeontology and archaeology
☒	☐	Animals and other organisms
☒	☐	Clinical data
☒	☐	Dual use research of concern
☒	☐	Plants

Methods

n/a	Involved in the study	
☒	☐	ChIP-seq
☒	☐	Flow cytometry
☒	☐	MRI-based neuroimaging

Antibodies

Antibodies used	Anti-BICD1 rabbit polyclonal, Atlas Antibodies, HPA041309, 1:200 dilution<br>Anti-BICD2 rabbit polyclonal, Atlas Antibodies, HPA023013, 1:200 dilution<br>GFP-Booster Alexa Fluor 488, alpaca nanobody, Chromotek, gb2AF488, 1:500 dilution<br>Anti-GAPDH mouse monoclonal, Abcam, ab8245, 1:1000 dilution<br>Anti-β-tubulin, mouse monoclonal, Sigma, T4026, 1:1000 dilution<br>Anti-TSA56, rat monoclonal, 1:200 dilution
Validation	All antibodies except for anti-TSA56 are commercially available and have been validated by manufacturers. Anti-TSA56 was custom made for the Salje lab and has been validated by the provider (Abclonal). Citation: https://pubmed.ncbi.nlm.nih.gov/35739103/

Eukaryotic cell lines

Policy information about cell lines and Sex and Gender in Research

Cell line source(s)	FLp-In T-Rex Cells - Thermofisher (R71407)<br>HeLa ATCC CCL-2 - ATCC (https://www.atcc.org/products/ccl-2)<br>Murine L929 cells - ATCC (CCL-1 https://www.atcc.org/products/ccl-1)<br>Lenti-X 293Ts Thermofisher (NC9834960).<br><br>We made the following cell lines in this study:<br>HeLa Flp-In T-REX cell lines (GFP, GFP-ScaA, GFP-ScaC, GFP-ScaD, GFP-ScaE, GFPMTS, GFP-BICD2NMTS and GFP-ScaCMTS)<br>HeLa ATCC CCL-2 cell lines (BICD1-/-, BICD2-/-, BICD1-/-&BIC2-/-)
Authentication	Cell lines were purchased directly from the vendors, but were not further authenticated.
Mycoplasma contamination	All cell lines tested negative for mycoplasma contamination
Commonly misidentified lines<br>(See ICLAC register)	No common misidentified cell lines were used in this study

Plants

Seed stocks	*Report on the source of all seed stocks or other plant material used. If applicable, state the seed stock centre and catalogue number. If plant specimens were collected from the field, describe the collection location, date and sampling procedures.*
Novel plant genotypes	*Describe the methods by which all novel plant genotypes were produced. This includes those generated by transgenic approaches, gene editing, chemical/radiation-based mutagenesis and hybridization. For transgenic lines, describe the transformation method, the number of independent lines analyzed and the generation upon which experiments were performed. For gene-edited lines, describe the editor used, the endogenous sequence targeted for editing, the targeting guide RNA sequence (if applicable) and how the editor was applied.*
Authentication	*Describe any authentication procedures for each seed stock used or novel genotype generated. Describe any experiments used to assess the effect of a mutation and, where applicable, how potential secondary effects (e.g. second site T-DNA insertions, mosiacism, off-target gene editing) were examined.*
